# An unusual case of metaphyseal osteonecrosis of humerus in a post covid patient: a case report

**DOI:** 10.11604/pamj.2022.42.244.34337

**Published:** 2022-07-29

**Authors:** Ajayakumar Thankappan, Nizaj Nasimudeen, Arun Thomas, Jyothi Karikkanthra, Jojo Pullockara

**Affiliations:** 1 Department of Orthopaedics, Apollo Adlux Hospital, Kochi, Kerala, India,; 2 Department of Radiodiagnosis, Apollo Adlux Hospital, Kochi, Kerala, India,; 3 Department of Pathology, Apollo Adlux Hospital, Kochi, Kerala, India,; 4 Department of Nephrology, Apollo Adlux Hospital, Kochi, Kerala, India

**Keywords:** COVID-19, osteonecrosis, pathological fracture, metaphysis, case report

## Abstract

Osteonecrosis of the metaphysis is often rare as it is a highly vascular region. Here we report an unusual case of non-traumatic osteonecrosis of the humerus predominantly involving the metaphysis in a post covid elderly female. The patient had a pathological fracture of humerus during the post-operative period of intertrochanteric femur fracture surgery. She was evaluated for the causes of pathological fracture and the fracture was managed with hemi replacement of the shoulder because of the extensive bone loss. The pathology here could only be explained as some sequelae of hyper inflammatory state associated with COVID-19 infection. The possible differentials are also discussed here. This case report will help clinicians to consider COVID-19 infection as a cause for non-traumatic osteonecrosis among other reported causes of osteonecrosis.

## Introduction

The rapidly spreading COVID-19 infection has been a major challenge especially in India with a high population density. The manifestations of the disease are still evolving and are under close scientific scrutiny. Recent evidences suggest that COVID-19 adversely affects different human body systems as a part of “long COVID-19”, such as Guillain-Barré syndrome, lung fibrosis, pulmonary thromboembolism, cardiomyopathy, sensory dysfunction and stroke [1]. The musculoskeletal symptoms, including myalgia, arthralgia, and fatigue, are a nearly constant presence from the mild to severe stages of COVID-19 disease [2]. The long term osteoarticular manifestations are yet to be quantitated. The large scale prolonged use of corticosteroids, prolonged immobilisation, hypercoagulable state of the infection can contribute to the musculoskeletal manifestations. Coagulopathy, thrombotic events and few reports of avascular necrosis of femoral head have been described in patients with COVID-19. However, osteonecrosis at the metaphyseal region are yet not reported. Here, we report an unusual case of osteonecrosis of the humerus predominantly involving the metaphysis in an elderly female.

## Patient and observation

**Figure 1:**
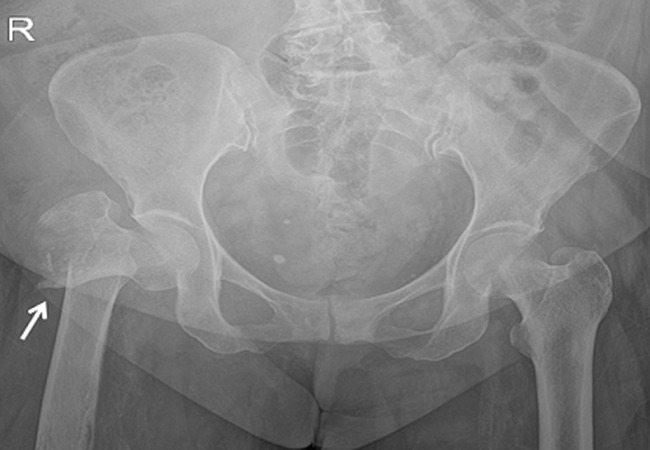
preoperative X-ray of pelvis showing displaced intertrochanteric fracture right femur

**Patient information**: sixty years old female, a known case of type 2 diabetes mellitus and hypertension admitted in a tertiary centre with fever and breathlessness 2 months prior to admission in our facility. She was diagnosed with SARS-CoV-2 (COVID-19) infection and was on IV steroids, Remdisivir and other supportive measures. She developed post covid pneumonia which later progressed to sepsis and acute kidney injury. High Resolution Computed Tomography (HRCT) chest was suggestive of post covid sequelae with superadded secondary infection. A bronchoscopy was done and the bronchoalveolar lavage showed the growth of Burkholderia pseudomallei. Blood culture revealed growth of B. pseudomallei, Vancomycin resistant enterococcus faecalis and Pseudomonas, for which culture specific IV antibiotics (Ceftazidime, Aztreonam, Doxycycline) were given. She was then referred to a local facility for continuation of IV antibiotics. During her stay in that hospital, she had a fall and sustained displaced intertrochanteric fracture of right proximal femur (Figure 1). She was referred to our facility for the management of the fracture and the worsening renal functions. On presentation to our hospital, she had acute renal injury (Urea (144 mg%), creatinine (5.06 mg%)) with near normal urine output.

**Clinical findings**: she had European Pressure Ulcer Advisory Panel (EPUAP) grade three bedsores over sacrum. Her right lower limb was shortened and externally rotated due to the fracture. The trochanteric femur fracture was managed with proximal femoral nailing (PFN A2).

**Timeline**: on 3^rd^ post-operative day (after roximal femur surgery), she complained of pain over her left shoulder and restriction of shoulder movements with no definite history of trauma to her shoulder. There was a mild swelling over the proximal arm with no skin discolouration or local rise of temperature. She was afebrile.

**Figure 2:**
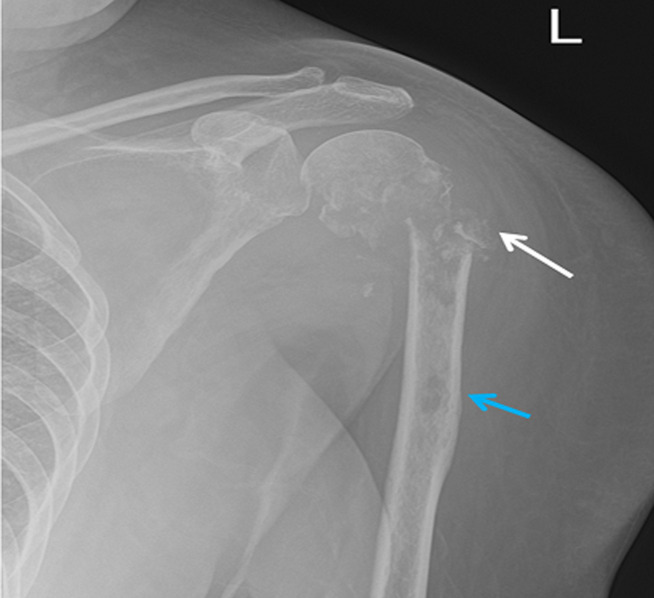
preoperative X-ray of left shoulder showing pathological comminuted fracture of proximal humerus (involving neck, greater and lesser tuberosities) with extensive lytic areas and cortical destruction of the metadiaphyseal region of the humerus (white arrow), and another intramedullary lytic area at the proximal diaphysis (blue arrow)

**Figure 3:**
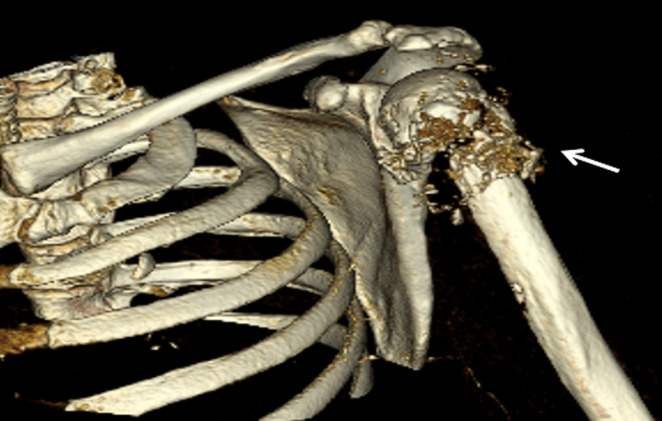
CT 3-D imaging of left shoulder showing pathological fracture of proximal left humerus with areas of lysis and cortical destruction

**Diagnostic approach**: a radiograph of the left shoulder and humerus (Figure 2) showed pathological comminuted fracture of proximal humerus (involving neck, greater and lesser tuberosities) with extensive lytic areas and cortical destruction of the metadiaphyseal region of the humerus. Another intramedullary lytic area was noted at the proximal diaphysis. Computed tomography (CT) scan of the left shoulder (Figure 3) confirmed the above findings of pathological fracture of proximal left humerus with areas of lysis and cortical destruction. No major collections or features of significant inflammation were seen in the adjacent soft tissue and muscles. Laboratory evaluation of blood showed: S calcium (7.7 mg%), S phosphorus (4.8 mg%), alkaline phosphatase (146 U/L), parathyroid hormone (+128pg/ml), Vitamin D (8 ng/ml), Hb (7.6gm %), Urea (174 mg %), creatinine (2.4 mg %), albumin (1.9 gm %), ESR (115 mm/hr), CRP (184 mg/L), cancer antigen ((CA -125) (28 U/ml (normal)), carcino embronic antigen ((CEA) 2.4 ng/ml (normal)), alpha fetoprotein (AFP) (2.5ng/ml (normal)). Serum electrophoresis showed no monoclonal bands.

**Figure 4:**
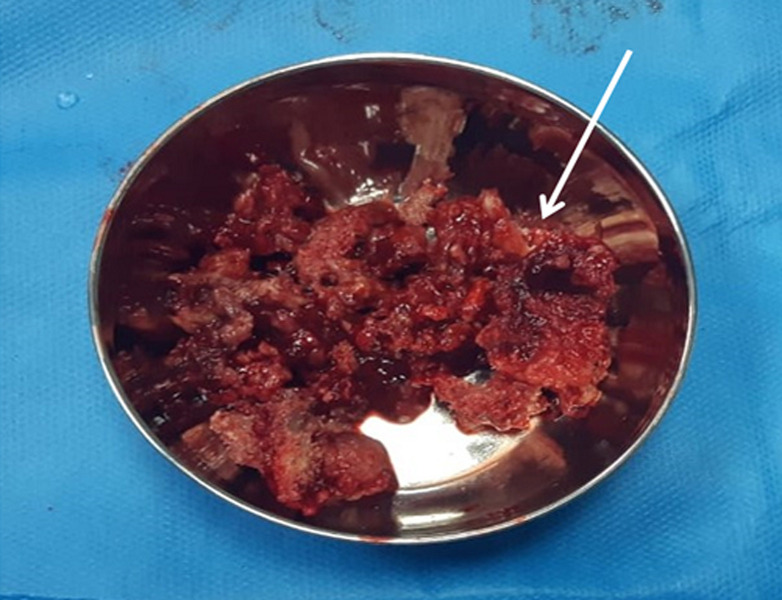
intraoperative specimen showing multiple bony fragments obtained from left proximal humerus pathological fracture site

**Figure 5:**
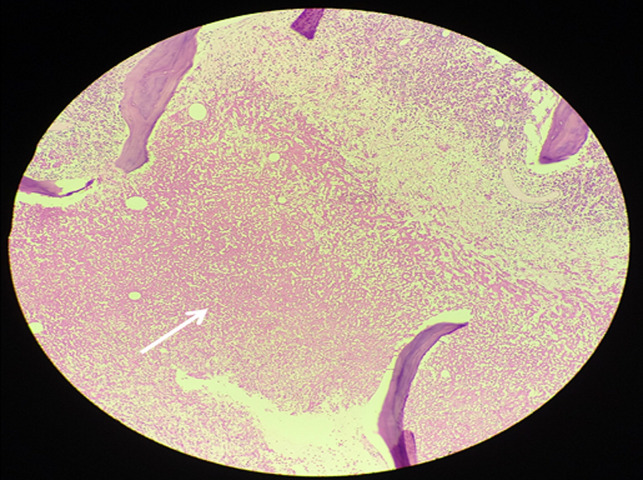
H & E stained section 40 x magnification showing extensive coagulative necrosis of marrow within intertrabecular spaces

**Therapeutic intervention**: considering the differentials of infection, malignancy, secondary hyperparathyroidism and melioidosis of bone, we planned for open biopsy and fixation of the humerus fracture through deltopectoral approach. Intraoperatively, the bone was found excessively friable without much soft tissue involvement or pus collection. Another unicortical lytic lesion was seen at the diaphysis which was further away from the metaphyseal lytic lesion. Since the bone was excessively friable and due to poor bone stock at the head and neck, osteosynthesis was abandoned and a cemented hemi replacement was done. The bone and tissue (Figure 4) were sent for culture and sensitivity as well as histopathological examination. The tissue and bone culture reports showed no growth of any organisms. Histopathological examination revealed extensive marrow necrosis without any signs of malignancy or infection (Figure 5). Postoperatively, the general condition of the patient improved and the wound healed well.

**Figure 6:**
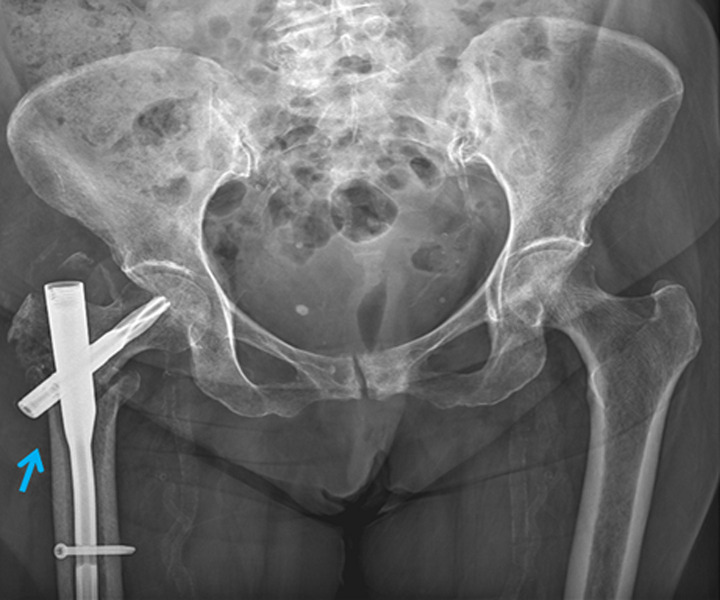
postoperative X-ray of pelvis at 6 months follow-up showing proximal femoral nail (PFN A2) on right side

**Figure 7:**
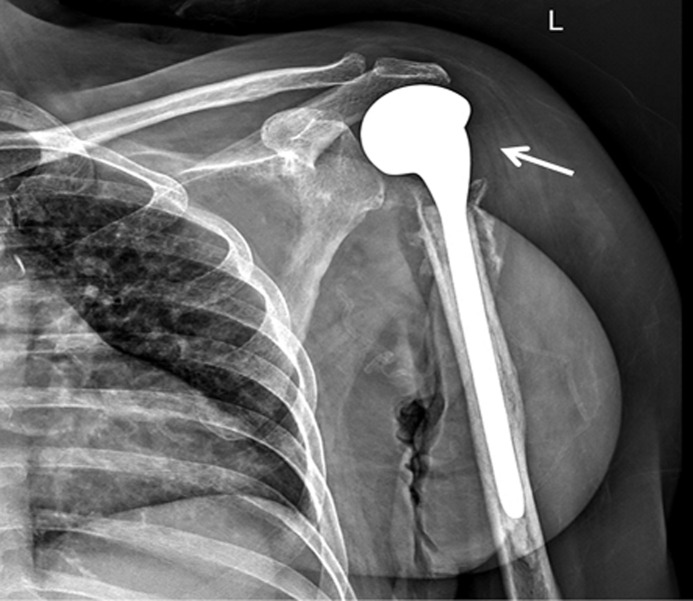
postoperative X-ray of left shoulder at 6 months follow-up showing hemi replacement implant in situ; there are no new lesions or any signs of implant loosening

**Follow-up**: with FDG PET scan showed no evidence of malignancy or distant metastasis. She had regular follow-up with X-rays (Figure 6, Figure 7) and blood investigations. There are no new lesions or any signs of implant loosening on follow-up radiographs. At the latest follow-up at 6 months, she is asymptomatic with normal lung and renal functions, and normal PTH levels.

**Consent**: we confirm that the patient has given informed consent for the case report to be published.

## Discussion

Osteonecrosis has been reported in various pathologies like pancreatitis, sickle cell disease, caisson´s disease and other hypercoagulable states [3]. The exaggerated inflammatory response of novel coronavirus infection (COVID-19) can lead to severe manifestations such as adult respiratory syndrome, sepsis, coagulopathy, and death in a proportion of patients. A coagulopathy has been reported in up to 50% of patients with severe COVID-19 manifestations [4]. Infection-induced endothelial dysfunction causes an excess of procoagulant thrombin with concomitant shutting down of the fibrinolytic cascade resulting in a hypercoagulable state which can spur thrombosis by increasing blood viscosity and coupled with hypoxia can activate the hypoxia-induced transcription factor-dependent signalling [5]. SARS-CoV-2 ORF3a protein and host hypoxia-inducible factor (HIF-1Þ) play key role in the virus infection and pro-inflammatory responses. The hypoxia-inducible factor 1Þ (HIF-1Þ) acts as a key regulator in physiological functions including metabolism, cell proliferation, and angiogenesis. HIF-1Þ up regulation of Sox 9 activity may also have a chondroprotective role following femoral head ischemia [6].

Interestingly, during SARS-CoV-2 infection, ORF3a induces Mito-ROS production to activate HIF-1Þ, which in turn enhances the viral infection and aggravates inflammatory responses [7]. The proposed theories behind pathogenesis of non-traumatic ON include intraosseus hypertension, intravascular fat or gaseous emboli and extravascular compression by increased marrow fat stores etc. Most support a “multiple hit” theory, with accumulated tissue stress from various insults reaching a critical threshold and initiating the disease process [3]. The metaphysis is a highly vascular area and osteonecrosis at the site is often rare. The pathology here can be best explained with the microvascular thrombosis associated with severe COVID-19 infection. The incidence of osteonecrosis in our case can be increased several fold with the associated comorbidities like diabetes and prolonged corticosteroid use. Other possibilities which we considered include malignancy, osteomyelitis, hyperparathyroidism. Culture and sensitivity of bone and soft tissue obtained during surgery did not show any growth of organisms. HPE showed extensive marrow necrosis and did not show any features of malignancy/osteomyelitis. PET scan and tumour markers were negative for malignancy. Primary hyperparathyoidism is ruled out by absence of parathyroid adenoma and absence of high levels of serum PTH.Secondary hyperparathyroidism leading to lytic bone lesion in this case was unlikely due to the very short span of kidney disease which returned to near normal levels with treatment.

## Conclusion

Based on the clinical, radiological, histopathological and microbiological analysis, we presume that the osteonecrosis involving the metaphysis of humerus in our case is predominantly due to COVID-19 induced hyper inflammatory response. Our case report will help clinicians to consider COVID-19 infection as a cause for non-traumatic osteonecrosis among other reported causes of osteonecrosis.
